# Analysis of sealing ability of endodontic cements apical plugs

**DOI:** 10.4317/jced.54186

**Published:** 2018-02-01

**Authors:** Bruna Cechella, Josiane de Almeida, Morgane Kuntze, Wilson Felippe

**Affiliations:** 1 DDS, MSc, Department of Endodontics, School of Dentistry, Federal University of Santa Catarina, Florianópolis, SC, Brazil; 2 DDS, MSc, PhD, Professor of Endodontics, University of Southern Santa Catarina, Palhoça, Brazil; 3 DDS, MSc, PhD student in Endodontics, Department of Endodontics, School of Dentistry, Federal University of Santa Catarina, Florianópolis, SC, Brazil; 4 DDS, MSc, PhD, Associate Professor of Endodontics, Department of Endodontics, School of Dentistry, Federal University of Santa Catarina, Florianópolis, SC, Brazil

## Abstract

**Background:**

It is unknown whether the bioactivity of Biodentine characterized by the precipitation of hydroxyapatite and formation of tags into the dentinal tubules improve its sealing ability as an apical plug. Aim: To evaluate the sealing ability provided by Biodentine and mineral trioxide aggregate (MTA) apical plugs, with or without phosphate-buffered saline (PBS) intracanal dressing, using a glucose leakage method.

**Material and Methods:**

The space of the canal of 100 root segments with about 12 mm long was shaped using Gates-Glidden. After created an apical retrograde cavity, the root segments were randomly divided into 4 groups (n = 25): G1 – Biodentine; G2 - Biodentine + PBS intracanal dressing; G3 - MTA and G4 - MTA + PBS intracanal dressing. All access openings were filled with temporary cement and all root segments were introduced in floral foams moistened with PBS. After 2 months, all root segments were prepared to evaluate the glucose leakage. The amount of glucose leakage was quantified by a spectrophotometer and the data were analyzed using chi-square test (*p*<0.05).

**Results:**

Traces of the glucose were observed in a higher of samples that received Biodentine apical plug (*p*<0.05). The exposure to intracanal PBS did not influence the sealing provided by Biodentine and MTA.

**Conclusions:**

The Biodentine had lower sealing ability than MTA. The interaction with PBS intracanal dressing did not improve the sealing ability provided by sealers.

** Key words:**Apexification, dental cements, dental leakage, glucose, pressure.

## Introduction

Mineral trioxide aggregate (MTA) has been used as apical plug in non-vital teeth with incompletely formed roots (1,2). In this situation, the marginal sealing provided by MTA is better compared to other materials (3,4), and ex vivo studies have demonstrated improvement when the cement remains in contact with the phosphate buffered saline (PBS) (5,6) due to the formation of carbonate apatite at the cement-dentin interface (7), with tag-like structures which partially obliterates the spaces between material and dentin (5).

Biodentine was introduced as an alternative to MTA. Based on tricalcium silicate (Ca3SiO5), this cement has also been suggested as apical plug. When its sealing ability was assessed by glucose leakage (8), it was demosntrated that Biodentine presents similar performance as the resin-modified glass ionomer cement (8).

Similar to MTA, its bioactivity has been evidenced by the precipitation of hydroxyapatite (9) and formation of tags that penetrate into the dentinal tubules (10,11). However, it is not known whether the sealing ability of Biodentine when used as an apical plug may be improved after interaction with PBS. Therefore, this study evaluated the sealing ability provided by Biodentine and MTA apical plugs, with or without phosphate-buffered saline (PBS) intracanal dressing, using a glucose leakage method. The null hypothesis was that the exposure of Biodentine and MTA to PBS would improve the sealing ability.

## Material and Methods

One hundred-four extracted, human, single-rooted teeth were used. The study was approved by the Ethics Committee for Research with Human Beings of the Federal University of Santa Catarina (protocol number 167.645).

The procedures were performed as described by Almeida, *et al.* (12). The crowns were sectioned, and a 2 mm root tip resection was performed with a high-speed bur under cooling water, so that all root segments were about 12 mm long. The canals were cleaned and shaped using #1-5 Gates-Glidden drills (Dentsply Malleifer, Ballaigues, Switzerland) in a crown-down fashion, and 1% sodium hypochlorite (NaOCl) (Asfer Indústria Química, São Caetano do Sul, SP, Brazil) was used for irrigation. A standardized open apex was created by retrograde preparation of the canal with a #6 Gates-Glidden drill (±1.50 mm diameter). The final canal rinse was performed with 17% EDTA (Asfer Indústria Química, São Caetano do Sul, SP, Brazil) followed by 1% NaOCl.

-Procedure for apical plug

One hundred root segments were randomly divided into four experimental groups, according to the cement used for fabrication of the apical plug and contact of the plug with PBS ( Table 1).

**Table 1:**
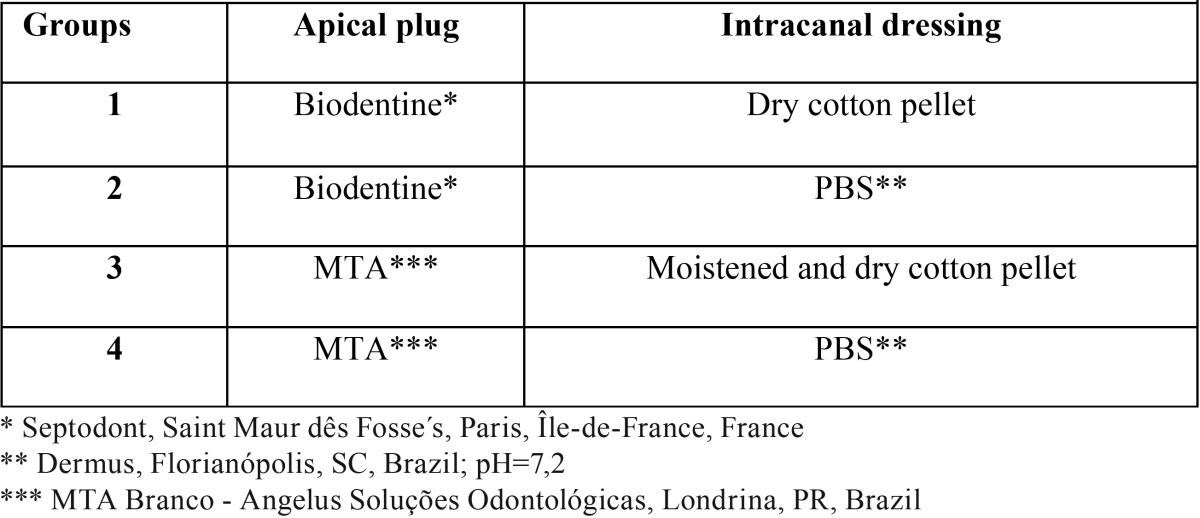
Groups and Materials.

MTA and Biodentine was mixed following the manufacturer’s recommendations. The cement mixture was introduced into the canal, condensed with paper points, and compacted with pluggers (Dentsply, Tulsa Dental,Tulsa, OK, USA) to create a 4 mm thick apical plug. Radiographs were taken from all root segments to ensure void-free MTA placement and plug thickness. In group 1, a dry cotton pellet was placed in the cervical region of each root segment. In groups 3, a cotton pellet moistened with distilled water was placed in the cervical region of each root segment, which was replaced by a dry pellet after 24 h. In groups 2 and 4, the remaining canal space was filled with PBS (Dermus Farmácia Dermatológica e Coméstica Ltda, Florianópolis, SC, Brazil; pH=7.2) as an intracanal dressing ( Table 1). All access openings were covered with cotton pellets and filled with temporary cement (Cimpat, Septodont Brasil Ltda, São Paulo, SP, Brazil). Thereafter, the root segments were introduced in plastic vials containing floral foam moistened with 20 mL PBS and stored for 2 months at 37°C. PBS solution was replaced every 5 days.

-Assembled double chamber and glucose leakage measuring

**Figure 1:**
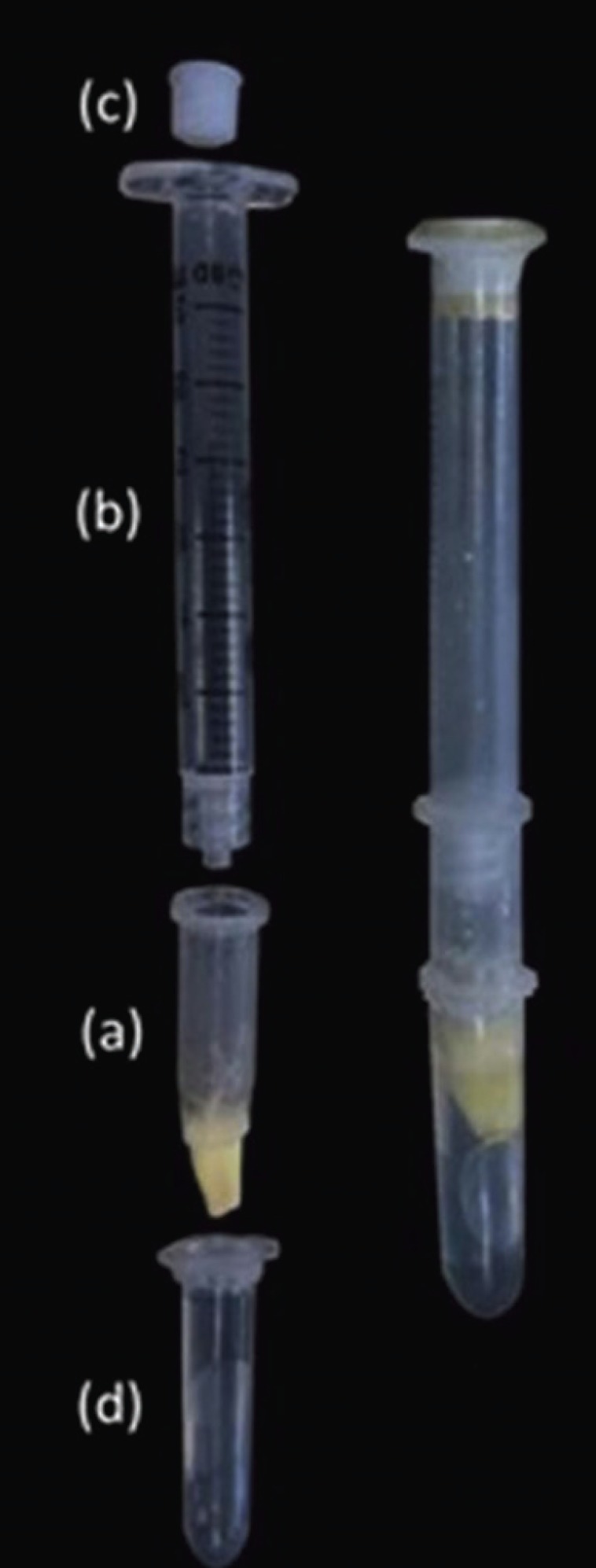
Photograph of part of the device developed for the glucose leakage test. (a) 1.5 mL Eppendorf tube with the root segment; (b) syringe; (c) screwed device; (d) 2.0 mL Eppendorf containing deionized water.

**Figure 2:**
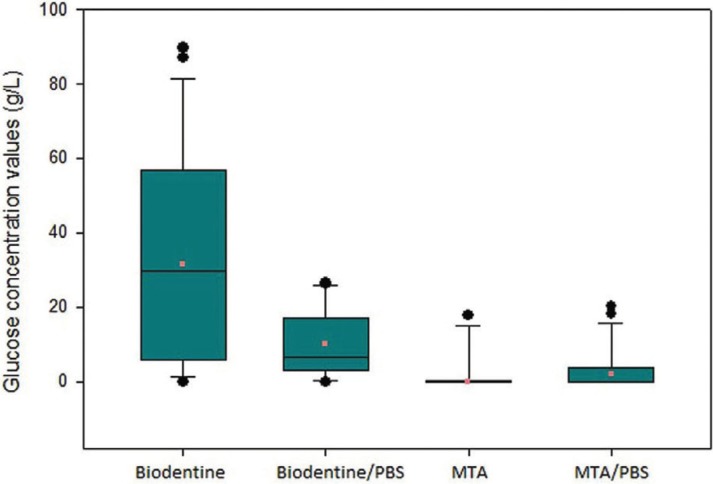
Box plots of the glucose concentration values in each group, illustrating the mean traces, minimal and maximal glucose traces and the median.

The root segments were fixed in a device designed to test glucose leakage [adapted from Leal, *et al.* (13) and Almeida, *et al.* (12) (Fig. 1). The cervical portion of each root segment was fastened in a 1.5 mL Eppendorf tube with the apical 7 mm protruding through the end (a). The upper portion of this Eppendorf tube was adapted a syringe (b) connected to a screwed device (c) through which 1.4 mL of 1 mol L-1 of glucose solution was injected. The lower portion of the Eppendorf, containing the root segment, was adapted inside a second 2.0 mL Eppendorf (d) containing 1.4 mL of deionized water, so that the apical 3 mm of the root were immersed in the water. Cyanoacrylate adhesive (Loctite Super Bonder, Henkel Ltda, São Paulo, SP, Brazil) was used to seal all interfaces and connections, (Fig. 2).

For the positive control group (n=2), root segments without apical plugs were used. Two teeth with intact crowns, to which two layers of nail varnish (Colorama, Procosa Produtos de Beleza Ltda, São Paulo, SP, Brazil) were applied over the root surface, were used as negative control group (n=2).

A pressure of 103 KPa (15 psi) was created by a compressed air pump (Inalar Compact, NS Indústria de Aparelhos Médicos, São Paulo, SP, Brazil), which was connected to a system constituted by a manometer, a valve to control the pressure and a cannula in which the screw device, connected to the syringe, was fixed. The glucose solution was forced into the tube for 60 min. A system was developed to run eight root segments simultaneously.

A 10 μL aliquot of solution contained in the Eppendorf (sample) was drawn using a micropipette, and traces of glucose were identified using a glucose kit (Glicose Pap Liquiform, Labtest Diagnóstica, Lagoa Santa, MG, Brazil).

Each sample was analyzed using a UV/VSI spectrophotometer (BIO-2000, Bioplus 2004R, Barueri, SP, Brazil) at 505 nm wavelength to obtain a specific optical density, and the values were converted to glucose concentration. All readings were taken in duplicate, and the mean value was considered for statistical analysis.

-Statistical analysis

The set of data, represented by the frequency of apical plugs presenting leakage in each group, was statistically analyzed by the chi-square test at a significance level of 5%.

## Results

In the negative control group no trace of glucose solution was detected, whereas in the positive control group the mean value of glucose concentration was 92.15 g/L.

The number and percentage of samples that showed traces of solution, as well as the mean value of glucose concentration, are shown in  Table 2.

**Table 2:**
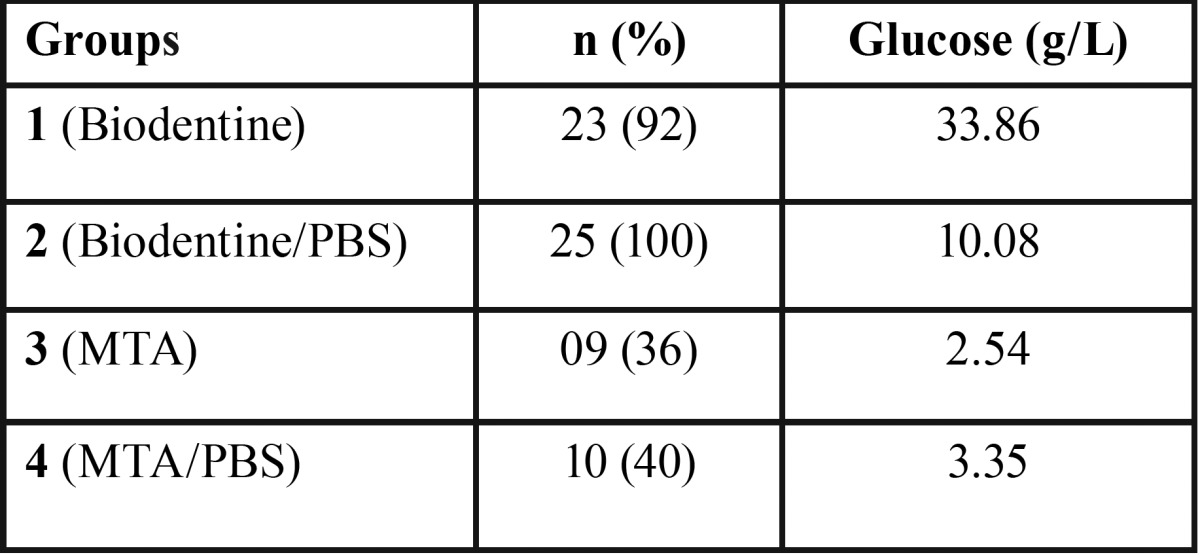
Groups, Number and Percentage of Samples with Traces of Glucose Solution and Concentration Mean Value.

Smaller number of specimens with traces of glucose (*p* < 0.05) were observed in root segments receiving the MTA apical plug (groups 3 and 4). The exposure to intracanal PBS did not influence the sealing provided by Biodentine (groups 1 and 2) (*p* > 0.05) and MTA (groups 3 and 4) (*p* > 0.05).

## Discussion

Several methods have been used to evaluate the sealing ability of endodontic materials (12-14). However, none of these methods is able to reproduce the situations observed *in vivo* (15).

The glucose leakage method proposed by Xu, *et al.* (16) presents high specificity and high sensitivity (16,17). This method has been used to evaluate the sealing ability of different materials (13,16,18), including MTA (12,13,19).

In this study, most root segments receiving an MTA apical plug did not allow leakage (n = 16/64%), therefore demonstrating satisfactory sealing ability and confirming the findings of previous studies (3,4). Conversely, glucose has been detected in more than 1/3 of specimens, corroborating the findings that MTA does not provide a completely efficient sealing (5,13,20-22).

Previous studies demonstrated that PBS positively influences the sealing provided by MTA (5,6), due to the formation of carbonate apatite at the cement-dentin interface (7), which partially obliterates the spaces between material and dentin (5). Different than expected and from the results of previous studies (5,6), yet confirming the findings of Almeida, *et al.* (12), this study demonstrated that the intracanal PBS did not significantly improve the sealing provided by the MTA apical plug. The occurrence of leakage has been explained by the presence of failures within the cement, at the cement-dentin interface (23) and/or by the presence of pores interconnected to MTA (24).

Most root segments receiving the Biodentine apical plug allowed glucose leakage (n = 23/92%). This unsatisfactory result may be related to the porosity of this material (25).

As also observed for MTA, the interaction between Biodentine/dentin and intracanal PBS did not improve the sealing ability of the cement. It is possible that calcium chloride (CaCl2) present in the Biodentine liquid may have influenced the outcomes. The incorporation of CaCl2 to a tricalcium silicate cement results in smaller quantity of water for material admixture (14). However, due to the hygroscopic action of calcium chloride (26), the contact of Biodentine with intracanal PBS may have allowed greater water absorption by the cement. This would cause change in the powder/liquid ratio, favoring the formation of many pores (27) in Biodentine, with direct implications on leakage. It may be assumed that, for this reason, a greater number of root segments with Biodentine/PBS presented leakage when compared to MTA/PBS.

The modified polycarboxylate may also have accounted for this occurrence (28). This polymer is a dispersant widely used in civil engineering, being classified as a super-plastifying agent. When mixed with water, the cement grains tend to uptake part of the liquid. Polycarboxylate, acting as a dispersant, prevents this uptake and assures greater fluidity to the cement. However, when in contact with PBS, it is possible that Biodentine has a greater quantity of water available, consequently presenting greater dispersion of particles (28), allowing the incorporation of air and facilitating the formation of pores.

Based on the present results, it was possible to conclude that, when used as apical plug, Biodentine presented worse sealing ability than MTA, and the use of intracanal PBS did not improve the sealing ability of these cements.
